# Optimal vitamin D spurs serotonin: 1,25-dihydroxyvitamin D represses serotonin reuptake transport (*SERT*) and degradation (*MAO-A*) gene expression in cultured rat serotonergic neuronal cell lines

**DOI:** 10.1186/s12263-018-0605-7

**Published:** 2018-07-11

**Authors:** Marya S. Sabir, Mark R. Haussler, Sanchita Mallick, Ichiro Kaneko, Daniel A. Lucas, Carol A. Haussler, G. Kerr Whitfield, Peter W. Jurutka

**Affiliations:** 1 School of Mathematical and Natural Sciences, Arizona State University, Phoenix, AZ USA; 2 Department of Basic Medical Sciences, University of Arizona College of Medicine, Phoenix, AZ USA

**Keywords:** Autism spectrum disorders, Depression, Neuropsychiatric disorders, Social behavior, Tryptophan metabolism, Vitamin D receptor, 5-Hydroxytryptamine

## Abstract

**Background:**

Diminished brain levels of two neurohormones, 5-hydroxytryptamine (5-HT; serotonin) and 1,25-dihydroxyvitamin D_3_ (1,25D; active vitamin D metabolite), are proposed to play a role in the atypical social behaviors associated with psychological conditions including autism spectrum disorders and depression. We reported previously that 1,25D induces expression of tryptophan hydroxylase-2 (TPH2), the initial and rate-limiting enzyme in the biosynthetic pathway to 5-HT, in cultured rat serotonergic neuronal cells. However, other enzymes and transporters in the pathway of tryptophan metabolism had yet to be examined with respect to the actions of vitamin D. Herein, we probed the response of neuronal cells to 1,25D by quantifying mRNA expression of serotonin synthesis isozymes, TPH1 and TPH2, as well as expression of the serotonin reuptake transporter (SERT), and the enzyme responsible for serotonin catabolism, monoamine oxidase-A (MAO-A). We also assessed the direct production of serotonin in cell culture in response to 1,25D.

**Results:**

Employing quantitative real-time PCR, we demonstrate that *TPH-1/-2* mRNAs are 28- to 33-fold induced by 10 nM 1,25D treatment of cultured rat serotonergic neuronal cells (RN46A-B14), and the enhancement of *TPH2* mRNA by 1,25D is dependent on the degree of neuron-like character of the cells. In contrast, examination of *SERT*, the gene product of which is a target for the SSRI-class of antidepressants, and *MAO-A*, which encodes the predominant catabolic enzyme in the serotonin pathway, reveals that their mRNAs are 51–59% repressed by 10 nM 1,25D treatment of RN46A-B14 cells. Finally, serotonin concentrations are significantly enhanced (2.9-fold) by 10 nM 1,25D in this system.

**Conclusions:**

These results are consistent with the concept that vitamin D maintains extracellular fluid serotonin concentrations in the brain, thereby offering an explanation for how vitamin D could influence the trajectory and development of neuropsychiatric disorders. Given the profile of gene regulation in cultured RN46A-B14 serotonergic neurons, we conclude that 1,25D acts not only to induce serotonin synthesis, but also functions at an indirect, molecular-genomic stage to mimic SSRIs and MAO inhibitors, likely elevating serotonin in the CNS. These data suggest that optimal vitamin D status may contribute to improving behavioral pathophysiologies resulting from dysregulation of serotonergic neurotransmission.

## Background

Serotonin, a neurotransmitter derived from the nutritionally essential amino acid tryptophan, executes critical functions in the brain such as control of appetite, energy expenditure, sleep, temperature, mood, and social cognition [1]. Serotonergic neurons innervate vast areas of the brain, with projections arising from cell bodies in the dorsal and median raphe and neighboring nuclei of the lower brain stem. These projections radiate to the hippocampus, amygdala, hypothalamus, nucleus accumbens, and lateral prefrontal cortex, as well as to a widespread array of cortical areas, wherein serotonin modulates a broad range of behavioral actions via metabotropic G protein-coupled and ionotropic, ligand-gated ion channel receptors [2].

In what constitutes the first step of serotonin production, tryptophan in the CNS is hydroxylated to 5-hydroxytryptophan by the enzyme tryptophan hydroxylase type 2 (TPH2), the rate-limiting step in brain serotonin synthesis. This is followed by subsequent decarboxylation of 5-hydroxytryptophan, catalyzed by aromatic amino acid decarboxylase, to serotonin (5-hydroxytryptamine; 5-HT). Serotonin is then packaged by vesicularization through the action of monoamine transporter isoform 2 in raphe neurons. After the release of 5-HT-containing vesicles from axon terminals, and subsequent reuptake of serotonin from the synapse via a serotonin reuptake transporter (SERT), degradation of serotonin is catalyzed by monoamine oxidase-A (MAO-A) and aldehyde dehydrogenase to the predominant serotonin metabolite, 5-hydroxyindoleacetic acid [2]. Therefore, three important enzymes/transport proteins that determine serotonin concentration and dynamics in the CNS are TPH2, SERT, and MAO-A. It is generally accepted that the amount and activity of these three proteins governs serotonin levels in the CNS [3].

In a recent publication [4], we proposed that serotonin concentrations are controlled in part by 1,25-dihydroxyvitamin D_3_ (1,25D), the active, hormonal metabolite of vitamin D. Specifically, we demonstrated that 1,25D dramatically induces *TPH2* gene expression in differentiated serotonergic rat raphe RN46A-B14 cells [4], a finding that is reinforced by in vivo experiments conducted by Jiang et al. [5] revealing a significant induction of *TPH2* mRNA in the prefrontal cortex of rats after chronic 1,25D administration. Central among the plethora of serotonin bioeffects are those that influence executive function, sensory gating, and prosocial behavior; importantly, serotonin levels are often suboptimal in disorders of these actions. Patrick and Ames [6] have reviewed the observed association between aberrant serotonin concentrations, both during development and in the adult, in a broad range of behavioral illnesses including ADHD, autism, bipolar disorder, depression, and schizophrenia, as well as anti-social, obsessive-compulsive, and suicidal behaviors. They hypothesized that an association between vitamin D insufficiency and low central serotonin concentrations represents a common denominator in a myriad of neuropsychiatric disorders. While it is conceivable that the ability of the vitamin D hormone, 1,25D, to upregulate *TPH2* expression is a key factor in maintaining serotonin output by serotonergic neurons, other avenues of serotonin metabolism have yet to be investigated with respect to the effects of vitamin D. In the present communication, we examined the influence of a range of 1,25D concentrations on the expression of *SERT*, *MAO-A/B*, and *TPH-1/2* to elucidate the mechanism of action underlying the regulation of serotonin reuptake, degradation, and synthesis by vitamin D in serotonergic neurons.

## Results

**Fig. 1:**
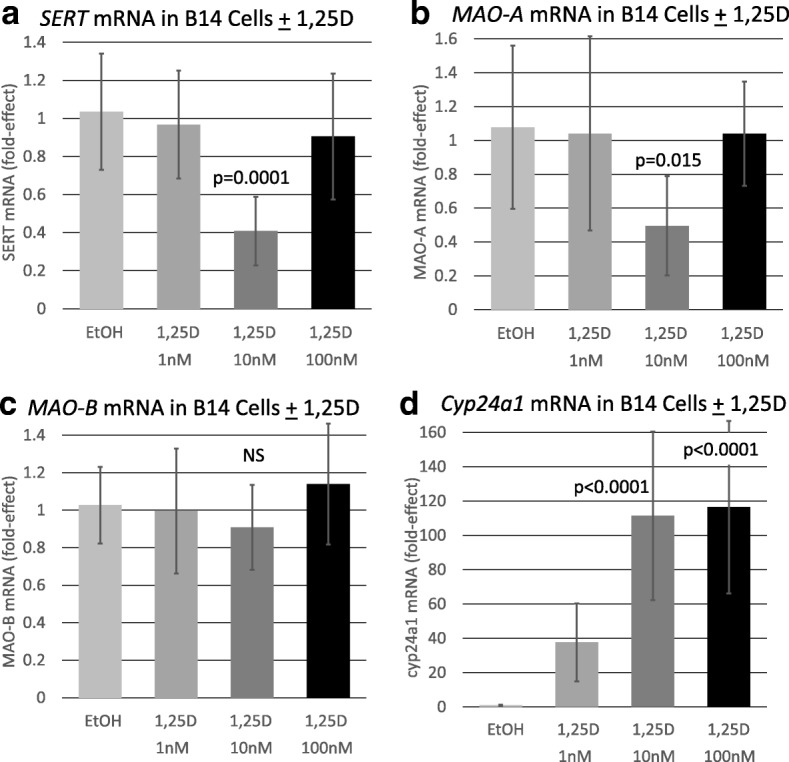
Effect of 1,25D on selected serotonin degrading pathway and control mRNAs in a serotonergic neuronal cell line. *P* values listed at the top of bars represent statistical significance for comparison between 1,25D treatment and ethanol vehicle control group in each and all cases. Statistical analyses were performed using ANOVA with a post hoc adjustment of *P* values via the Dunnett test that compares every mean to a control mean and takes into account the scatter of all the groups. **a** Expression of *SERT* in serotonergic rat raphe RN46A-B14 cells treated with 1,25D. Each mRNA value is the average of nine biological replicates ± std. dev. **b** Expression of *MAO-A* in serotonergic rat raphe RN46A-B14 cells treated with 1,25D. Each mRNA value is the average of nine biological replicates ± std. dev. **c** Expression of *MAO-B* in serotonergic rat raphe RN46A-B14 cells treated with 1,25D. Each mRNA value is the average of 18 biological replicates ± std. dev. **d** Expression of *Cyp24a1* in serotonergic rat raphe RN46A-B14 cells treated with 1,25D. Each mRNA value is the average of nine biological replicates ± std. dev

To determine if vitamin D affects the major route of serotonin reuptake and degradation, the influence of 1,25D on the expression of *SERT* and *MAO-A* was assessed by quantitative real-time PCR in differentiated serotonergic rat raphe RN46A-B14 cells. Cells in culture were challenged with 1, 10, and 100 nM 1,25D, and mRNA was isolated, reverse transcribed to cDNA and probed for *SERT* and *MAO-A* expression. Figure 1a illustrates that *SERT* is unaffected by 1 and 100 nM 1,25D-treatment, but a concentration of 10 nM 1,25D significantly (*p* = 0.0001) represses *SERT* mRNA expression by 59%. Thus, the response of *SERT* to 1,25D is biphasic, reminiscent of the U-shaped curves generated when plotting circulating 25-hydroxyvitamin D levels versus disease and death in humans [7]. Strikingly, *MAO-A* displays an identical regulatory pattern of expression when challenged with a range of 1,25D concentrations in differentiated serotonergic rat raphe RN46A-B14 cells (Fig. 1b). *MAO-A* is similarly unaffected by 1 and 100 nM 1,25D-treatment, but a concentration of 10 nM 1,25D significantly (*p* = 0.015) represses *MAO-A* mRNA expression by 51%. Therefore, two neurally expressed genes exhibit a similar profile of regulation by 1,25D, and the data suggest that certain levels of the vitamin D hormone appear to be capable of suppressing serotonin reuptake and catabolism.

As a negative-control neural gene, *MAO-B* was evaluated for modulation of expression by 1,25D in RN46A-B14 cells (Fig. 1c). Interestingly, *MAO-B* was not significantly repressed by 1,25D at any of the tested dosages, indicating that the influence of 1,25D on *SERT* and *MAO-A* expression may be selective for biologically relevant serotonin metabolism genes. Furthermore, as a positive control to confirm endogenous expression of a sufficient copy number of biologically functional nuclear vitamin D receptors (VDRs) to mediate transcriptional regulation by 1,25D in RN46A-B14 cells, we examined *Cyp24a1* mRNA enhancement in response to the vitamin D hormone. As depicted in Fig. 1d, *Cyp24a1* expression is dramatically (38- to 116-fold) augmented by exposure of the cells to 1–100 nM 1,25D, with the pertinent observation that this gene displayed the traditional dose-response curve for induction. *Cyp24a1* serves not only as a control for positive modulation of gene expression via VDR in RN46A-B14 cells, but because the RNA probed was isolated from the same treated cells for *SERT* and *MAO-A* mRNA assessment, any systematic error in the technology can be eliminated from consideration as an explanation for the unique dose-response results for serotonin catabolic gene expression.

**Fig. 2:**
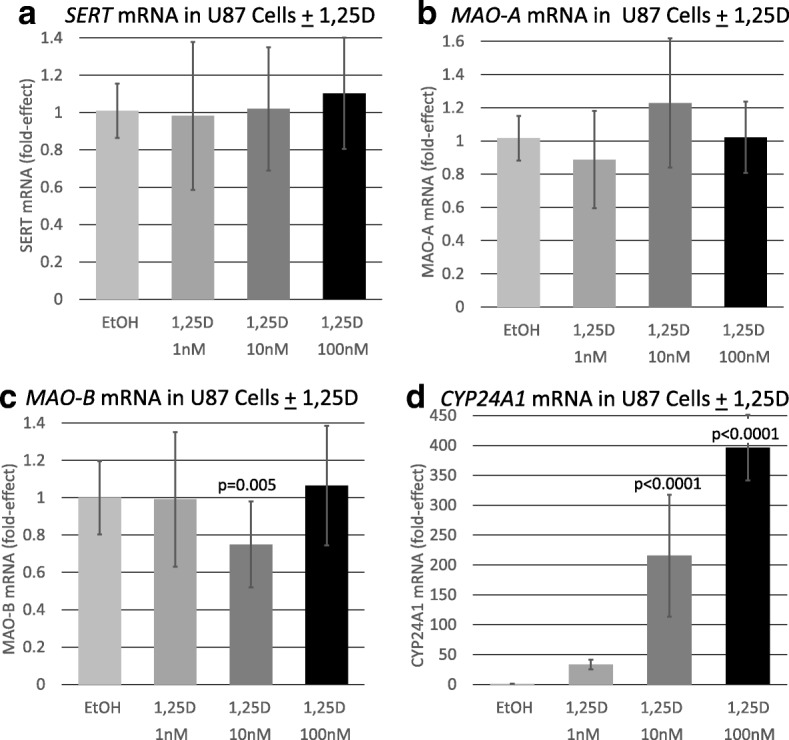
Effect of 1,25D on serotonin degrading pathway and control mRNAs in U87 glioblastoma cells. *P* values listed at the top of bars represent statistical significance for comparison between 1,25D-treatment and ethanol vehicle control group in each and all cases. Statistical analyses were performed using ANOVA with a post hoc adjustment of *P* values via the Dunnett test that compares every mean to a control mean and takes into account the scatter of all the groups. **a** Expression of *SERT* in U87 human glioblastoma cells treated with 1,25D. Each mRNA value is the average of seven biological replicates ± std. dev. **b** Expression of *MAO-A* in U87 human glioblastoma cells treated with 1,25D. Each mRNA value is the average of 11 biological replicates ± std. dev. **c** Expression of *MAO-B* in U87 glioblastoma cells treated with 1,25D. Each mRNA value is the average of 27 biological replicates ± std. dev. **d** Expression of *CYP24A1* in U87 glioblastoma cells treated with 1,25D. Each mRNA value represents the average of seven biological replicates ± std. dev

Next, we determined if 1,25D exerted actions on the expression of *SERT*, *MAO-A*, *MAO-B*, and *CYP24A1* in the human glioblastoma cell line, U-87 MG (U87), which does not exhibit serotonergic neuronal characteristics. In contrast to the results in RN46A-B14 cells, the data for U87 cells reveal that 1,25D does not significantly alter the expression of either *SERT* or *MAO-A* in glioblastoma cells (Fig. 2a, b). This observation suggests that the repressive modulation of these two serotonin-degradative reactions by 1,25D may be specific for serotonergic neurons. Further, as depicted in Fig. 2c, unlike in RN46A-B14 cells, 1,25D significantly represses (25%, *p* = 0.005) *MAO-B* mRNA levels in U87 cells, pointing to a potential selectivity of 1,25D action on the MAO-B isozyme in regions of the CNS separate from serotonergic neurotransmission. Interestingly, the same biphasic pattern of U-shaped dose-responsiveness to 1,25D obtained for *SERT* and *MAO-A* repression in RN46A-B14 cells occurs for *MAO-B* in U87 cells in terms of the optimal repressive dose of 1,25D being 10 nM. That this finding is valid is supported by the classic dose-response relationship occurring for 1,25D induction of *CYP24A1* in U87 cells (Fig. 2d), which serves as a positive control.

**Fig. 3:**
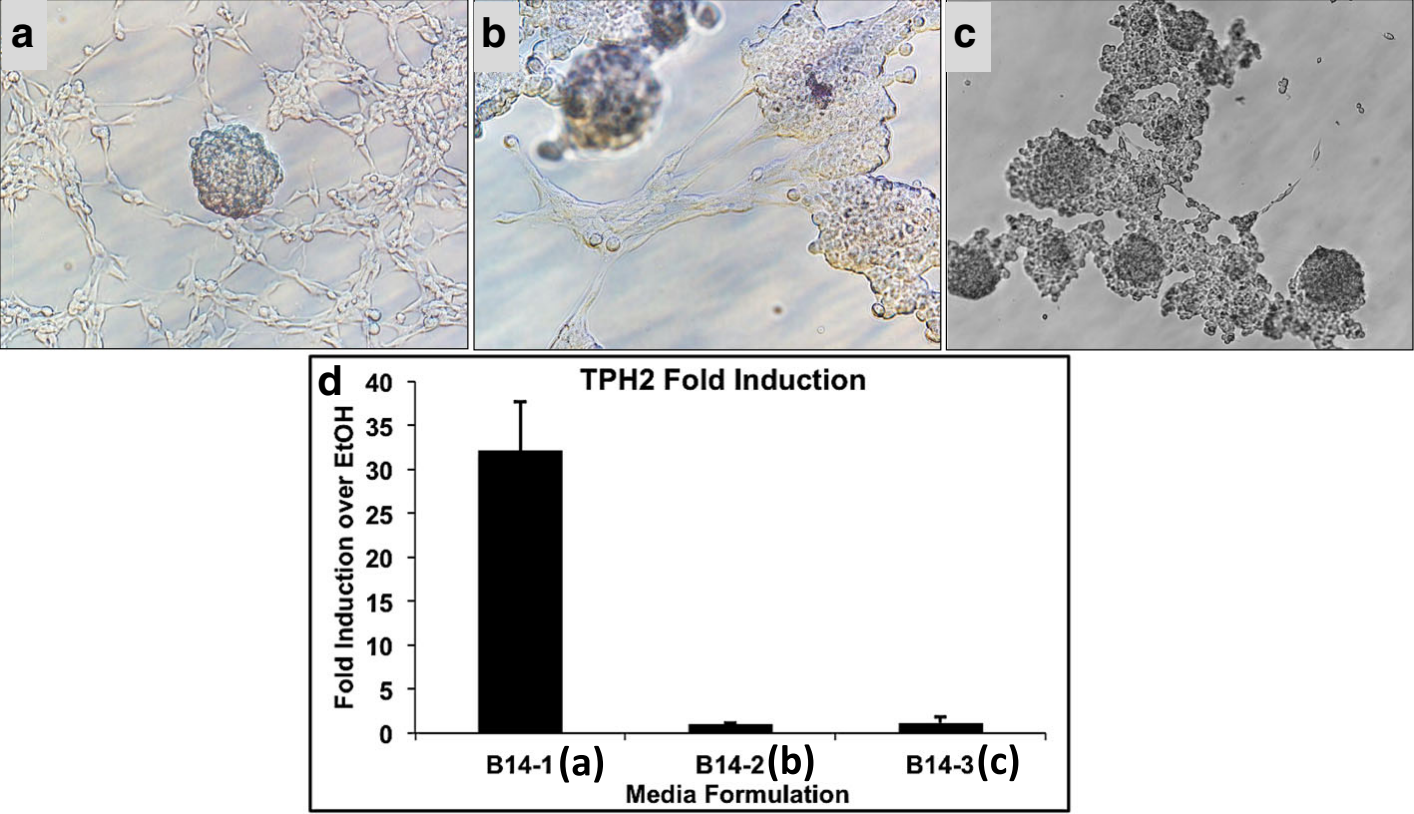
Morphology of serotonergic rat raphe RN46A-B14 cells grown in **a** media 1, **b** media 2, and **c** media 3, see the “Methods” section for descriptions of the media formulations. **d** RN46A-B14 cells grown in media 1–3 and treated with 10 nM 1,25D for 24 h, followed by *TPH2* mRNA quantification, expressed as fold relative to EtOH vehicle control (not shown)

In order to investigate the influence of serotonergic neuronal cell state on the control of serotonin synthesis by 1,25D, we next studied RN46A-B14 cells cultured in three different media formulations designed to generate varying degrees of neuronal morphology. The three media, designated media 1, 2, and 3, are described in detail in the “Methods” section. Figure 3a pictorially represents RN46A-B14 cells grown in media 1, illustrating their fusiform stellate morphology and neurite interconnections; cells maintained in media 1 displayed the most neuronal-like morphology and exhibited the slowest growth rate among the tested media. Figure 3b shows RN46A-B14 cells grown in media 2, with the cells retaining generally neuronal morphology, but acquiring some fibroblast-like character with increased growth rate and confluency. RN46A-B14 cells maintained in media 3 (Fig. 3c) displayed mostly fibroblast morphology during proliferation, with the fastest growth rate, likely induced by components of fetal bovine serum (FBS) and additional glucose, both present in the media. Therefore, media 1 elicited the most neuron-like character in RN46A-B14 cells, and the cells assumed a more fibroblast-like morphology and proliferated more rapidly as the media progressed from 1 to 3. Figure 3d depicts the results of *TPH2* mRNA induction by treatment with 10 nM 1,25D for 24 h of RN46A-B14 cells maintained in media 1–3. *TPH2* was significantly (32-fold) induced by 1,25D only in media 1, revealing that 1,25D regulation of tryptophan metabolizing gene products occurs in cells most resembling serotonergic neurons.

**Fig. 4:**
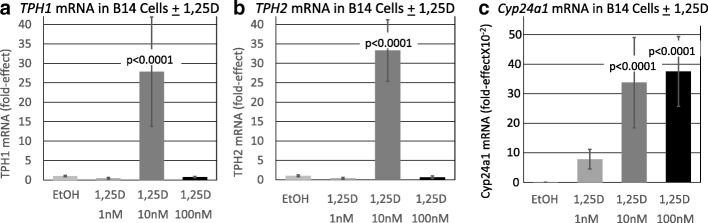
Effect of 1,25D on *TPH1, TPH2* and *Cyp24a1* mRNAs in a serotonergic neuronal cell line. *P* values listed at the top of bars represent statistical significance for comparison between 1,25D-treatment and ethanol vehicle control group in each and all cases. Statistical analyses were performed using ANOVA with a post hoc adjustment of *P* values via the Dunnett test that compares every mean to a control mean and takes into account the scatter of all the groups. **a** Expression of *TPH1* in serotonergic rat raphe RN46A-B14 cells treated with 1,25D. The exact fold-effect values for 1 and 100 nM 1,25D are too small to be perceived in the figure as scaled; they are 0.41 ± 0.22 and 0.73 ± 0.17, respectively. Each mRNA value is the average of nine biological replicates ± std. dev. **b** Expression of *TPH2* in serotonergic rat raphe RN46A-B14 cells treated with 1,25D. The exact fold-effect values for 1 and 100 nM 1,25D are too small to be perceived in the figure as scaled; they are 0.36 ± 0.23 and 0.65 ± 0.31, respectively. Each mRNA value is the average of 30 biological replicates ± std. dev. **c** Expression of *Cyp24a1* in serotonergic rat raphe RN46A-B14 cells treated with 1,25D. Each mRNA value is the average of 20 biological replicates ± std. dev

Next, the concentration-dependence of 1,25D-regulation of both *TPH1* and *TPH2* mRNAs was evaluated in RN46A-B14 cells maintained in media 1. As illustrated in Fig. 4a, the profile of *TPH1* mRNA response to 1,25D as a function of concentration of the vitamin D hormone is analogous to the results obtained for *SERT* and *MAO-A* but inverted, in that treatment of the cells with 1 or 100 nM 1,25D is without significant effect whereas exposure of the cells to 10 nM 1,25D induces *TPH1* 28-fold*.* Again, this biphasic induction over a range of hormone concentrations is not the consequence of experimental error because when assayed in RNA from the same biological replicate cells, *Cyp24a1* mRNA exhibits the traditional dose-response curve for induction following treatment of the cells with 1,25D (Fig. 4c). This non-traditional inverted U-shaped dose response is addressed in the “Discussion” section, but at present the authors postulate that specific, CNS-expressed genes may be modulated in their expression differently than are typical vitamin D target genes in peripheral tissues such as bone, intestine, and kidney. Consistent with this interpretation is the 1,25D concentration-dependence of regulation of *TPH2* mRNA in RN46A-B14 cells (Fig. 4b). TPH2 is documented to be the isoform that is responsible for catalyzing brain serotonin synthesis in the adult [8]. In non-neuronal serotonergic cells, TPH1 mRNA is the primary isoform expressed, whereas serotonergic neurons express TPH2 mRNA predominantly [9]. As depicted in Fig. 4b, the profile of *TPH2* mRNA response to 1,25D as a function of concentration of the vitamin D hormone mimics the pattern of regulation of *TPH1*, in that treatment of the cells with 1 or 100 nM 1,25D produces no significant effect on *TPH2* mRNA, whereas exposure of the cells to 10 nM 1,25D induces *TPH2* 33-fold (*p* < 0.0001)*.* What could be important with respect to serotonin dynamics is that the inverted U-shaped curve for *TPH2* induction by 1,25D as a function of concentration of hormone (Fig. 4b) is complementary to the U-shaped curves for *SERT* (Fig. 1a) and *MAO-A* (Fig. 1b) mRNA repression in response to increasing 1,25D concentration.

**Fig. 5:**
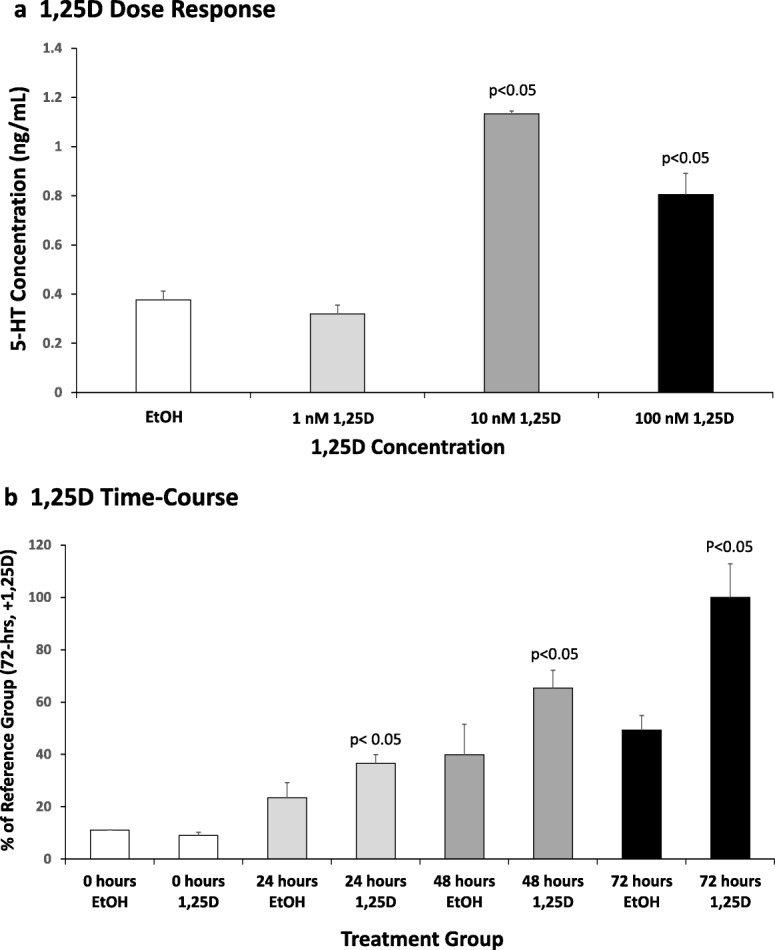
Effect of 1,25D on total 5-HT levels in rat serotonergic neuron cell culture medium. **a** A dose-response experiment was performed in serotonergic rat raphe RN46A-B14 cells treated with the indicated concentrations of 1,25D or ethanol (EtOH) vehicle. A 200 μL sample of medium was removed from each plate after 72 h of treatment and 5-HT quantitated by ELISA as detailed in the “Methods” section. Values are the average of three biological replicates (*n* = 3) ± SD, with duplicate or triplicate samples averaged in each group. **b** A time-course experiment was carried out in RN46A-B14 cells treated with the optimal concentration of 1,25D (10 nM) for 0, 24, 48, and 72 h followed by removal of a 200 μL sample of medium from each plate and serotonin assayed by 5-HT ELISA. Values are the average of three biological replicates (*n* = 3) ± SD, with duplicate or triplicate sample groups averaged in each case

In a final set of in vitro experiments, RN46A-B14 cells were exposed to 1, 10, or 100 nM 1,25D for 72 h and serotonin concentrations were quantified by 5-HT ELISA of the cell culture medium. Interestingly, 1 nM 1,25D exerted a slight (15%), non-significant suppression of serotonin compared to ethanol vehicle control, which contrasted to the 2.9-fold increase (*p* < 0.05) in serotonin induced by 10 nM 1,25D (Fig. 5a). Remarkably, considering that this assay is cumulative, Fig. 5a also illustrates that 100 nM 1,25D treatment of RN46A-B14 cells produced a diminished induction of serotonin to 2.0-fold, generating a biphasic dose-response curve for total serotonin that reflects the biphasic dose-response curves for the induction of *TPH2* as well as for the repression of *SERT* and *MAO-A*. Taken together, this stimulatory effect of 10 nM 1,25D on serotonin synthesis, coupled with suppressive actions on serotonin reuptake and metabolic elimination, resulting in almost a tripling of total free serotonin, vividly integrates what a significant effect an optimal concentration of 1,25D (10 nM) exerts to bolster extracellular serotonin. Figure 5b displays the time-course of serotonin enhancement by 10 nM 1,25D in RN46A-B14 cell culture. Augmented serotonin is apparent at 24 h (1.7-fold; *p* < 0.05), levels off at 48 h (1.6-fold; *p* < 0.05), and reaches 2.0-fold (*p* < 0.05) by 72 h of exposure to 1,25D versus control ethanol vehicle. Such a time-course is consistent with induction/repression but does not distinguish between primary and secondary mechanisms.

## Discussion

Although recent research has highlighted the possible significance of the relationship between vitamin D and the tryptophan-derived neurotransmitter, serotonin [4], previous studies focused solely on serotonin synthesis. In the present communication, we have investigated, for the first time, the effects of 1,25D on the regulation of serotonin reuptake via a serotonin reuptake transporter and on the first step of serotonin degradation by monoamine oxidase-A in serotoninergic neuronal cell lines. SERT (SLC6A4, 5HTT), the 5-HT transporter, plays a crucial role in the control of serotonergic neurotransmission by determining the magnitude and duration of the 5-HT synaptic signal and has been an attractive target in rational neuropsychiatric drug design [10]. Fluoxetine (Prozac), citalopram (Celexa), paroxetine (Paxil), and sertraline (Zoloft) represent selective inhibitors of SERT. In the presence of these selective serotonin reuptake inhibitors (SSRIs), 5-HT remains in the extracellular space for an extended duration, allowing prolonged 5-HT receptor activation in the postsynaptic neuron. Thus, monoamine transporters such as SERT constitute the primary mechanism for terminating neurotransmission through removal of the released transmitter from the synaptic cleft. These transporters, including SERT, function as oligomeric complexes in the cell membrane, and are regulated primarily through phosphorylation, protein-protein interactions, and trafficking events [11]. Limited data are available on the control of *SERT* at the transcriptional level, although the genes encoding mammalian forms have been cloned [12]. The 5-prime flanking region of the *SERT (5HTT)* human gene contains a cyclic AMP-responsive element (CRE) at − 99 bp plus binding sites for AP-1, AP-2, and SP1, all just upstream of a TATA-like motif. Sequences within − 1.4 kb of the transcription start site confer cell-specific expression, and the basal promoter responds to induction by cAMP [12]. A functional polymorphism (5HTT-LPR) has been identified in the human *5HTT* promoter [13], and evidence has accumulated for an association between alleles of this variation and anxiety traits, affective disorders, as well as autism. Notably, three ChIP-seq databases generated in human immune cells for 1,25D-dependent occupation of vitamin D-responsive elements (VDREs) by VDR yield apparently functional sites at − 64.5 and + 118 kb in relation to the transcription start site of the *SERT* gene [14–16]. Thus, 1,25D could potentially modulate human *SERT* expression in a primary fashion via remote VDREs.

Examining the influence of 1,25D on *SERT* mRNA expression in rat raphe RN46A-B14 cells, we observed a repression of *SERT* mRNA by 59% when the cells were treated with 10 nM 1,25D (Fig. 1a). This repression was selective for serotonergic neuronal cells in that it was not found in U87 human glioblastoma cells (Fig. 2a). The initial conclusion is that 1,25D, analogous to SSRIs, suppresses serotonin reuptake, possibly potentiating serotonergic neurotransmission. However, this conclusion must be reached with caution, because neither 1 nor 100 nM 1,25D treatment of RN46A-B14 cells significantly represses *SERT* mRNA expression (Fig. 1a), generating an unusual, biphasic dose-response curve. Nevertheless, this dose response is reminiscent of the U-shaped curves generated when plotting circulating 25-hydroxyvitamin D levels versus disease in humans, most recently reported in a study of autism risk in 3-year-old Chinese children as a function of circulating 25-hydroxyvitamin D levels at birth [17]. In other words, there appears to exist an optimal level of circulating vitamin D for health, and this may correspond empirically to 10 nM 1,25D in the case of RN46A-B14 cell treatment.

However, the cellular and molecular mechanism that accounts for the observed effects of various concentrations of 1,25D in RN46A-B14 cell treatment experiments requires careful consideration. The authors favor the concept that limited, but more physiologically relevant, concentrations of 1,25D signal biologic actions such as cell proliferation, whereas higher levels of the hormone cause the opposing responses, namely inhibition of cell growth and apoptosis. We first identified this phenomenon when studying osteosarcoma cells in culture, observing that low concentrations of 1,25D stimulated cell growth, whereas higher levels drastically curbed proliferation [18]. Therefore, we propose that the data in Fig. 1a can be explained if one assumes that the minimum effective concentration to stimulate proliferation (and other effects such as altered transcription factor profile and epigenetic modifications) in RN46A-B14 cells is 10 nM 1,25D, conditioning the neuronal cells to exhibit a repression of *SERT* expression, then the lack of response to treatment with 1 nM is appropriate. If one then assumes that 100 nM 1,25D, analogous to the action of this level in many cell types, promulgates an inhibition of cell proliferation with attending alteration of transcription factor profile and epigenetic modifications, then the lack of responsiveness of *SERT* expression to this higher dose of 1,25D of these growth-inhibited RN46A-B14 cells is also readily explainable. Nevertheless, the observations, in vitro, that higher concentrations of 1,25D are less effective than 10 nM hormone must be viewed with some skepticism and it will ultimately need to be determined if data from cell lines can be directly equated to the U-shaped dose-response curves, in vivo, because cell growth aspects and cell culture conditions (i.e., interaction with other media components) could be responsible for the results with high 1,25D concentrations. However, very recent data obtained in autistic children suggest that maintaining circulating 25-hydroxyvitamin D levels in an optimal range elicits maximal therapeutic efficacy in vitamin D-responsive children [19]. Regardless of the mechanistic nuances, we provide herein more support for the vitamin D-serotonin hypothesis in the pathophysiology of neuropsychiatric disease that has been professed by Patrick and Ames [1, 6]. Consistent with the relevance of SERT modulation by 1,25D is a report [20] of increased concentrations of SERT protein (monitored by positron emission tomography (PET) brain scans) in patients with seasonal affective disorder (SAD). SERT levels in SAD patients were 5% higher in winter, when vitamin D generated by sunlight is deficient, than in summer when sunlight is at a maximum; control subjects without SAD manifest no change in SERT levels with season. It is accepted that sunlight is mood-elevating, and the 1,25D effect to repress SERT reported herein may represent a facet of this benefit.

When examining MAO-A modulation by 1,25D in rat raphe RN46A-B14 cells, we observed a repression of *MAO-A* mRNA by 51% when the cells were treated with 10 nM 1,25D (Fig. 1b). The 1,25D dose-response profile was equivalent for *MAO-A* and *SERT* repression, and the effect on *MAO-A* was also selective for serotonergic neuronal cells as it was not found in U87 human glioblastoma cells (Fig. 2b). Thus, the same arguments can be applied in discussing the control of *MAO-A* by 1,25D that were elaborated above for *SERT* repression. However, there exists a caveat to the observed repression of *MAO-A* by 1,25D in cultured rat serotonergic cells, in that Jiang et al. [5] demonstrated a significant induction of *MAO-A* mRNA in the prefrontal cortex of rats after chronic 1,25D administration. This conflict between the in vitro and in vivo results with respect to MAO-A could be because Jiang et al. [5] analyzed the entire prefrontal cortex, whereas the cell culture data are selective to serotonergic neuronal cells. Notably, MAO-A also catalyzes the degradation of dopamine and norepinephrine, as well as serotonin, implying a broader array of functions beyond serotonin catabolism in the cortex, perhaps explaining the differences between cell culture results and those obtained in rodent models.

*MAO-A* is a gene of interest that has been given the moniker “warrior gene” as it has been linked to aggression in observational and survey-based studies [21]. In addition, *MAO-A* knockout mice are more aggressive than their wild-type littermates or mice null for *MAO-B*, which degrades a different set of neurotransmitters [22]. The transcriptional regulation of *MAO-A* has not been fully characterized but, in a recent computational and experimental analysis of *Mao-A* expression, Gupta et al. [23] identified critical roles for a conserved proximal promoter domain (− 71/− 40 bp) in *cis*, and transcription factors Sp1/Gata2/Tbp in *trans*, to govern mouse *Mao-A* gene basal expression in a coordinated manner. We have located, in silico, a near-consensus candidate VDRE, consisting of a direct repeat with a spacer of three nucleotides (DR3), CGGACActgAGGTCA (antisense), at + 776 in relation to the start of transcription of the human *MAO-A* gene. Furthermore, Tuoresmaki et al. [16] have reported two VDR ChIP-seq target regions (roughly 200 bp in size) in the human *MAO-A* gene, the first of which overlaps the candidate DR3 VDRE at + 776 bp cited above and the second, located approximately 350 bp 3-prime to the first, which contains two additional candidate (antisense)VDREs: GGTGCAggaGGTTCT and GGTGCAggaGGTGCT. It is not yet known whether these putative VDREs are involved in *MAO-A* control by 1,25D, but their proximity to the core regulatory module at − 71/− 40 bp is suggestive of a possible *cis* regulatory module spanning the proximal promoter and first exon of the *MAO-A* gene. If this concept is verified, yet another avenue may exist whereby 1,25D could not only benefit patients with behavioral pathophysiologies resulting from dysregulation of serotonergic neurotransmission, but also impact other behavioral traits and neurological disorders, including Alzheimer’s dementia, aggression, and attention deficit hyperactivity disorder [23].

The present results support the vitamin D-serotonin hypothesis both experimentally and mechanistically. In fact, when we assessed the direct production of serotonin in neuronal cell culture in response to treatment with 10 nM 1,25D for 72 h, serotonin concentrations were significantly enhanced (two- to threefold) in the medium (Fig. 5), consistent with the concerted induction of *TPH2* (Fig. 4b) and repression of *SERT* (Fig. 1a) and *MAO-A* (Fig. 1b) by the empirically optimum level of 1,25D in cultured serotonergic neurons. Taking all of these results together, it seems highly likely that vitamin D can amplify serotonin in the central nervous system, making vitamin D a candidate for the treatment of neuropsychiatric disorders in which vitamin D and/or serotonin are implicated. Indeed, vitamin D supplementation has been reported to improve inattention, hyperactivity, and impulsivity in children and adults with ADHD [24].

## Conclusions

**Fig. 6:**
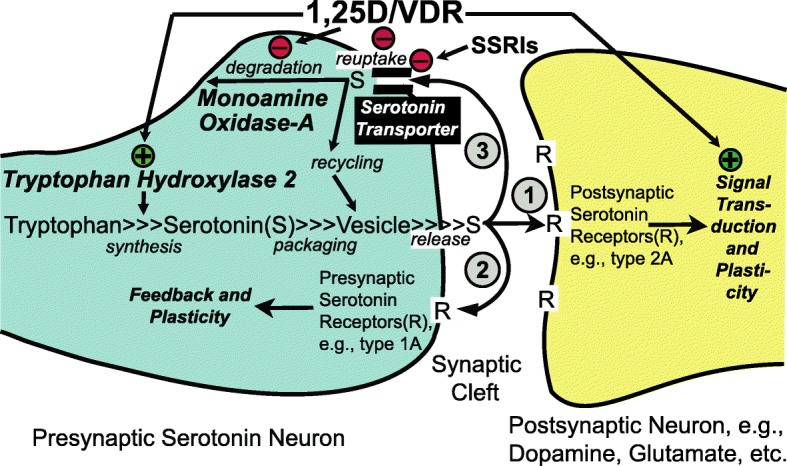
Actions of 1,25D/VDR on serotonin dynamics in the CNS at the serotonin synapse. This model depicts the fine-tuning of serotonin levels by 1,25D via transcriptional regulation of *TPH2*, *SERT*, and *MAO-A*; the result of which is the potentiation of serotonergic nerve transmission. Initially, (left) 1,25D induces *TPH2* to enhance serotonin output and, because circled pathway 3 (reuptake and degradation) is attenuated by 1,25D via repression of *SERT* and *MAO-A*, serotonin action is spurred at both presynaptic (circled pathway 2) and postsynaptic (circled pathway 1) sites through type 1A and 2A serotonin receptors, respectively

The results reported herein, combined with our previous work and that of Patrick and Ames [1, 6], lead us to conclude that a set of tryptophan metabolism pathway enzymes/proteins relevant to CNS levels of serotonin are transcriptionally regulated by 1,25D in differentiated serotonergic rat raphe RN46A-B14 cells in such a fashion as to suggest that optimum levels of the vitamin D hormone represent a natural effector in spurring serotonin. Strikingly, the modulation of the tryptophan metabolism pathway by 1,25D at the transcriptional level is conserved to the degree that it is even prominent in a vertebrate only distantly related to mammals, namely developing zebrafish [25]. By potentially fine-tuning serotonin concentrations in the synaptic cleft, as depicted schematically in the model in Fig. 6, 1,25D may be able to steer neurological control of such processes as social behavior to prevent autism spectrum disorders and depression. In summary, as illustrated in hypothetical form in Fig. 6, we conclude that the vitamin D hormone may be capable of governing serotonin concentrations in relevant regions of the brain where both *VDR* and *TPH2*, as well as *SERT* and *MAO-A*, are expressed, placing this nutrient at the forefront of nutrigenomic efforts to guide gene expression in serotonergic neurons toward ameliorating neuropsychiatric disease by spurring the actions of serotonin.

## Methods

### Mammalian cell culture

Two mammalian cell lines were employed in this study: embryonic rat medullary raphe (RN46A-B14; obtained from Dr. Robert J. Handa/Dr. John Neumaier) cultured with neurobasal A (Gibco/Life Technologies, Grand Island, NY) medium with B-27 supplement (Invitrogen Corporation) and l-glutamine (Gibco) and human brain glioblastoma/astrocytoma (U-87 MG, U87; obtained from the American Type Culture Collection) grown in DMEM/high glucose + l-glutamine + pyruvate (Hyclone, Logan, Utah). All cells were passaged in the above-indicated media supplemented with 10% FBS and penicillin/streptomycin (pen-strep) under a humidified atmosphere of 5% CO_2_ in air at 37 °C. RN46A-B14 cells in culture were utilized in their native state, i.e., not transfected with any expression vector. However, U87 cells were transfected with a VDR overexpression plasmid to ensure that they consistently expressed biologically functional levels of the nuclear receptor. The PolyJET transfection reagent (SignaGen, Rockville, MD) was utilized to transfect U87 cells according to the manufacturer’s protocol. Briefly, each well was transfected with 15 μL PolyJET reagent and 500 ng of pSG5-hVDR (plasmid-expressing human VDR).

The clonal cell line, RN46A-B14, was isolated following transfection of the gene encoding rat brain-derived neurotrophic factor (BDNF) into RN46A cells [26]. RN46A-B14 cells synthesize and secrete biologically active BDNF in vitro and synthesize serotonin following partial membrane depolarization. To optimize the growth conditions and neuronal morphology of adherent, rat serotonergic B14 cells in culture, three different media formulations were examined: media 1: neurobasal A medium, B-27 supplement, l-glutamine, and pen-strep (serum free); media 2: media 1 plus 10% FBS; media 3: DMEM/high glucose + l-glutamine + pyruvate, 10% FBS, and pen-strep.

### Quantitative real-time PCR

U87 and RN46A-B14 cells were plated at 500,000–800,000 cells per well in a 6-well plate, and dosed with 1–100 nM 1,25D (see Figs. 1, 2, 3 and 4) or ethanol vehicle control for 24 h. Total RNA was isolated from each well using an Aurum Total RNA Mini kit (Bio-Rad, Hercules, CA) according to the manufacturer’s instructions. The RNA quantity and quality were assessed using A_260/280_ spectrophotometry. DNase-treated RNA (1 μg) was reverse transcribed using an iScript cDNA Synthesis kit (Bio-Rad) to produce 20 μL of first-strand cDNA. For real-time PCR, 2 μL of cDNA was used in a 10 μL PCR reaction containing 5 μL FastStart Universal SYBR Green Master Mix + Rox (Roche Applied Science, Indianapolis, IN) and forward and reverse primers. Reactions were performed in 96-well PCR plates in a Bio-Rad CFX96 instrument using a standard 40-cycle profile. Data were analyzed using the comparative ΔΔCt method as means of relative quantitation, normalized to an endogenous reference (GAPDH) and relative to a calibrator (normalized Ct value from vehicle-treated cells) and expressed as 2^-ΔΔCt^ according to Applied Biosystems’ User Bulletin 2: Rev. B, “Relative Quantitation of Gene Expression.” Primer sets for the PCR were as follows:

Rat *GAPDH* (forward 5′-AGGTCGGTGTGAACGGATTTG-3′, reverse 5′-CATTCTCAGCCTTGACTGTGC-3′)

Rat *Cyp24a1* (forward 5’-AACGAAGCCTACGGGTTGATG-3′, reverse 5’-AGAAAGTCAGCCAAGACCTCA-3′)

Rat *SERT* [27] (forward 5′-TCTGAAAAGCCCCACTGGACT-3′, reverse 5′-TAGGACCGTGTCTTCATCAGGC-3′)

Rat *MAOA* [28] (forward 5′-CCACAGCCAGAGCGTTCAG-3′, reverse 5′-TGAGAGCCTTTGCCCAGATTG-3′)

Rat *MAOB* [28] (forward 5′-CAGTGGAAGCAGAGGAGAG-3′, reverse 5′-TGCTGCCATACCTGAGATG-3′)

Rat *TPH1* [29] (forward 5′-GCTGAACAAACTCTACCCAAC-3′, reverse 5′-TTCCCGATAGCCACAGTATT-3′)

Rat *TPH2* [30] (forward 5′-CTCCAAGCTTCGCATCACAG-3′, reverse 5′-AGCACTTCAGGAAGCGTACC-3′)

Human *GAPDH* (forward 5′-ACAACTTTGGTATCGTGGAAGGAC-3′, reverse 5′-CAGGGATGATGTTCTGGAGAGC-3′)

Human *CYP24A1* (forward 5′-CAGCGAACTGAACAAATGGTCG-3′, reverse 5′-TCTCTTCTCATACAACACGAGGCAG-3′)

Human *SERT* [31] (forward 5′-TTCTCCCCTCCAAGTGAATG-3′, reverse 5′-GGGGAATCCATGGAAATAAG-3′)

Human *MAOA* [32] (forward 5′-TAAATGGTCTCGGGAAGGTG-3′, reverse 5′-CCCAGGGCAGTTACTGATGT-3′)

Human *MAOB* [33] (forward 5′-GCTCTCTGGTTCCTGTGGTATGTG-3′, reverse 5′-TCCGCTCACTCACTTGACCAGATC-3′)

### Serotonin ELISA

To measure serotonin (5-HT) concentrations, colorimetric competitive 5-HT enzyme-linked immunosorbent assay (ELISA) experiments were performed utilizing the Serotonin Research ELISA kit (Rocky Mountain Diagnostics, Inc., Colorado Springs, CO). RN46A-B14 were plated at 700,000 cells per well in a six-well plate in media 2 (see above for details) and incubated in a humidified atmosphere of 5% CO_2_ at 37 °C overnight. The following day, media 2 was aspirated and the cells were gently washed with PBS to remove residual serum. Media 1 (see above for details) was added, and the cells were incubated overnight to generate a serum-deprived cell state. Next, the cells were treated with either ethanol vehicle control or 10 nM 1,25D for 0–72 h in media 1. Samples of media/culture supernatant (200 μL) were removed from each well (without disrupting the adherent cells) at 0, 24, 48, or 72 h and assayed for 5-HT content in accordance with the manufacturer’s protocol. Briefly, the diluent, wash buffer, six standards, and two positive controls were prepared at the time of the assay, and 100 μL of the diluted standards, controls, and experimental samples were acylated for 30 min at room temperature. Next, 100 μL of the acylated standards, controls, and experimental samples were placed into the appropriate wells of the serotonin/5-HIAA microtiter strips, and 25 μL of the 5-HT antiserum were added to each well and incubated for 15–20 h at 4 °C. The wells were washed, and 100 μL of the enzyme conjugate were added and allowed to incubate for 30 min followed by additional washes and incubation with 100 μL of the substrate. After the final incubation, 100 μL of the stop solution were added and the absorbance was read within 10 min using a microplate reader set to 450 nm and a reference wavelength between 620 and 650 nm. The calibration curve was obtained by plotting the absorbance readings measured for the standards (linear, *y* axis) against the corresponding standard concentrations (linear, *x* axis, ng/mL). A logarithmic trendline was fitted to the data for the standards and the corresponding equation was utilized to determine the serotonin concentrations in the experimental samples. The mean of three biological replicates, each with duplicate treatment groups, was utilized in the analysis.

### Statistical analysis

Data are expressed as means ± SD. All data are presented as fold-effects, with transcription of the gene in question (i.e., mRNA level) in the absence of 1,25D (i.e., ethanol vehicle) set at 1.0-fold. Thus, all results are normalized to basal transcription and presented graphically and numerically as fold-effect of the single tested variable, i.e., 1,25D. Because the design of the experiments was a simple motif in which the fold-ability of 1,25D to enhance transcription over basal (i.e., ethanol vehicle) was assessed, statistical differences between two groups were determined initially by a two-tailed Student’s *t* test. However, because variance was high in several experiments, further statistical analyses were performed using GraphPad Prism 7 to generate ANOVA data, followed by a post hoc Dunnett test that compares every mean to a control mean and takes into account the scatter of all the groups. In each figure illustrating the fold-effect of 1,25D, exact *P* values (unless < 0.0001) are listed above the bar for concentrations of 1,25D versus EtOH control, except when the difference from the vehicle control is not significant (NS); a *P* value less than 0.05 is considered significant.

## Abbreviations

1,25D: 1,25-Dihydroxyvitamin D_3_
5-HT: 5-Hydroxytryptamine
5-HTT: 5-HT transporter
DR3: Direct repeat with a spacer of 3 nucleotides
MAO-A: Monoamine oxidase-A
MAO-B: Monoamine oxidase-B
PET: Positron emission tomography
SAD: Seasonal affective disorder
SERT: Serotonin reuptake transporter
SSRI: Selective serotonin reuptake inhibitor
TPH1: Tryptophan hydroxylase-1
TPH2: Tryptophan hydroxylase-2
VDR: Vitamin D receptor
VDRE: Vitamin D-responsive element

## References

[1] Patrick RP, Ames BN (2014). Vitamin D hormone regulates serotonin synthesis. Part 1: relevance for autism. FASEB J.

[2] Jenkins TA, Nguyen JC, Polglaze KE, Bertrand PP (2016). Influence of tryptophan and serotonin on mood and cognition with a possible role of the gut-brain Axis. Nutrients.

[3] Andrews PW, Bharwani A, Lee KR, Fox M, Thomson JA (2015). Is serotonin an upper or a downer? The evolution of the serotonergic system and its role in depression and the antidepressant response. Neurosci Biobehav Rev.

[4] Kaneko I, Sabir MS, Dussik CM, Whitfield GK, Karrys A, Hsieh JC, Haussler MR, Meyer MB, Pike JW, Jurutka PW (2015). 1,25-Dihydroxyvitamin D regulates expression of the tryptophan hydroxylase 2 and leptin genes: implication for behavioral influences of vitamin D. FASEB J.

[5] Jiang P, Zhang LH, Cai HL, Li HD, Liu YP, Tang MM, Dang RL, Zhu WY, Xue Y, He X (2014). Neurochemical effects of chronic administration of calcitriol in rats. Nutrients.

[6] Patrick RP, Ames BN (2015). Vitamin D and the omega-3 fatty acids control serotonin synthesis and action, part 2: relevance for ADHD, bipolar disorder, schizophrenia, and impulsive behavior. FASEB J.

[7] Keisala T, Minasyan A, Lou YR, Zou J, Kalueff AV, Pyykko I, Tuohimaa P (2009). Premature aging in vitamin D receptor mutant mice. J Steroid Biochem Mol Biol.

[8] Zhang X, Beaulieu JM, Sotnikova TD, Gainetdinov RR, Caron MG (2004). Tryptophan hydroxylase-2 controls brain serotonin synthesis. Science.

[9] Gutknecht L, Kriegebaum C, Waider J, Schmitt A, Lesch KP (2009). Spatio-temporal expression of tryptophan hydroxylase isoforms in murine and human brain: convergent data from Tph2 knockout mice. Eur Neuropsychopharmacol.

[10] Linden DR, White SL, Brooks EM, Mawe GM (2009). Novel promoter and alternate transcription start site of the human serotonin reuptake transporter in intestinal mucosa. Neurogastroenterol Motil.

[11] Torres GE, Gainetdinov RR, Caron MG (2003). Plasma membrane monoamine transporters: structure, regulation and function. Nat Rev Neurosci.

[12] Heils A, Teufel A, Petri S, Seemann M, Bengel D, Balling U, Riederer P, Lesch KP (1995). Functional promoter and polyadenylation site mapping of the human serotonin (5-HT) transporter gene. J Neural Transm Gen Sect.

[13] Flattem NL, Blakely RD (2000). Modified structure of the human serotonin transporter promoter. Mol Psychiatry.

[14] Handel AE, Sandve GK, Disanto G, Berlanga-Taylor AJ, Gallone G, Hanwell H, Drablos F, Giovannoni G, Ebers GC, Ramagopalan SV (2013). Vitamin D receptor ChIP-seq in primary CD4+ cells: relationship to serum 25-hydroxyvitamin D levels and autoimmune disease. BMC Med.

[15] Heikkinen S, Vaisanen S, Pehkonen P, Seuter S, Benes V, Carlberg C (2011). Nuclear hormone 1alpha,25-dihydroxyvitamin D3 elicits a genome-wide shift in the locations of VDR chromatin occupancy. Nucleic Acids Res.

[16] Tuoresmaki P, Vaisanen S, Neme A, Heikkinen S, Carlberg C (2014). Patterns of genome-wide VDR locations. PLoS One.

[17] Wu D-M, Wen X, Han X-R, Wang S, Wang Y-J, Shen M, Fan S-H, Zhuang J, Li M-Q, Hu B, et al. Relationship between neonatal vitamin D at birth and risk of autism spectrum disorders: the NBSIB study. J Bone Miner Res. 2018;33(3):458–66. 10.1002/jbmr.3326 29178513

[18] Dokoh S, Donaldson CA, Haussler MR (1984). Influence of 1,25-dihydroxyvitamin D_3_ on cultured osteogenic sarcoma cells: correlation with the 1,25-dihydroxyvitamin D_3_ receptor. Cancer Res.

[19] Jia F, Shan L, Wang B, Li H, Feng J, Xu Z, Saad K. Fluctuations in clinical symptoms with changes in serum 25(OH)vitamin D levels in autistic children: three cases report. Nutr Neurosci. 2018; Epub ahead of print April 8 10.1080/1028415X.2018.1458421 29629638

[20] Mc Mahon B, Andersen SB, Madsen MK, Hjordt LV, Hageman I, Dam H, Svarer C, da Cunha-Bang S, Baare W, Madsen J (2016). Seasonal difference in brain serotonin transporter binding predicts symptom severity in patients with seasonal affective disorder. Brain.

[21] McDermott R, Tingley D, Cowden J, Frazzetto G, Johnson DD (2009). Monoamine oxidase A gene (MAOA) predicts behavioral aggression following provocation. Proc Natl Acad Sci U S A.

[22] Shih JC, Chen K (1999). MAO-A and -B gene knock-out mice exhibit distinctly different behavior. Neurobiology (Bp).

[23] Gupta V, Khan AA, Sasi BK, Mahapatra NR (2015). Molecular mechanism of monoamine oxidase A gene regulation under inflammation and ischemia-like conditions: key roles of the transcription factors GATA2, Sp1 and TBP. J Neurochem.

[24] Rucklidge JJ, Frampton CM, Gorman B, Boggis A (2014). Vitamin-mineral treatment of attention-deficit hyperactivity disorder in adults: double-blind randomised placebo-controlled trial. Br J Psychiatry.

[25] Craig TA, Zhang Y, McNulty MS, Middha S, Ketha H, Singh RJ, Magis AT, Funk C, Price ND, Ekker SC (2012). Research resource: whole transcriptome RNA sequencing detects multiple 1alpha,25-dihydroxyvitamin D(3)-sensitive metabolic pathways in developing zebrafish. Mol Endocrinol.

[26] Eaton MJ, Whittemore SR (1996). Autocrine BDNF secretion enhances the survival and serotonergic differentiation of raphe neuronal precursor cells grafted into the adult rat CNS. Exp Neurol.

[27] Abumaria N, Rygula R, Havemann-Reinecke U, Ruther E, Bodemer W, Roos C, Flugge G (2006). Identification of genes regulated by chronic social stress in the rat dorsal raphe nucleus. Cell Mol Neurobiol.

[28] Birgner C, Kindlundh-Hogberg AM, Oreland L, Alsio J, Lindblom J, Schioth HB, Bergstrom L (2008). Reduced activity of monoamine oxidase in the rat brain following repeated nandrolone decanoate administration. Brain Res.

[29] Charoenphandhu J, Teerapornpuntakit J, Nuntapornsak A, Krishnamra N, Charoenphandhu N (2011). Anxiety-like behaviors and expression of SERT and TPH in the dorsal raphe of estrogen- and fluoxetine-treated ovariectomized rats. Pharmacol Biochem Behav.

[30] Castro B, Sanchez P, Torres JM, Ortega E (2013). Effects of adult exposure to bisphenol a on genes involved in the physiopathology of rat prefrontal cortex. PLoS One.

[31] Bernard R, Kerman IA, Meng F, Evans SJ, Amrein I, Jones EG, Bunney WE, Akil H, Watson SJ, Thompson RC (2009). Gene expression profiling of neurochemically defined regions of the human brain by in situ hybridization-guided laser capture microdissection. J Neurosci Methods.

[32] Zhao H, Nolley R, Chen Z, Reese SW, Peehl DM (2008). Inhibition of monoamine oxidase a promotes secretory differentiation in basal prostatic epithelial cells. Differentiation.

[33] Jiang H, Jiang Q, Liu W, Feng J (2006). Parkin suppresses the expression of monoamine oxidases. J Biol Chem.

